# The forgotten risk? A systematic review of the effect of reminder systems for postpartum screening for type 2 diabetes in women with previous gestational diabetes

**DOI:** 10.1186/s13104-015-1334-2

**Published:** 2015-08-26

**Authors:** Charlotte Jeppesen, Jette Kolding Kristensen, Per Ovesen, Helle Terkildsen Maindal

**Affiliations:** Department of Public Health, Aarhus University, Bartholins Allé 2, 8000 Aarhus, Denmark; Department of Obstetrics and Gynaecology, Aarhus University Hospital, Aarhus, Denmark

**Keywords:** Postpartum follow-up, Type 2 diabetes, Gestational diabetes mellitus, Glucose screening, Reminder

## Abstract

**Background:**

Screening for type 2 diabetes is recommended for women with previous gestational diabetes (GDM). However, the screening rates remain low. We aimed to evaluate the reminders and reminder systems for women with previous GDM and the health professionals in primary and secondary health care with screening rate among postpartum women as primary outcome.

**Methods:**

Observational and intervention studies were included and the PRISMA guidelines were followed for the literature extraction.

**Results:**

Six studies were included: two long-term follow up studies and four early terms. Five studies focused on secondary care settings and one on primary care. Three studies focused on reminders to postpartum women only, two studies to both the women and health care professional, and one study on the health care provider only. Types of reminders varied from letters, emails, and personal telephone calls to the women to register-based reminders or letters to the health care professionals. Reminders were efficient but efficiency varied between studies. Two studies found that direct telephone calls strengthened the reminding of the women. The effect of reminding both the women and the health professional screening rates decreased compared to reminding either health professionals or reminding the women separately.

**Conclusions:**

Reminders have a potential for early detection and prevention of type 2 diabetes in this high risk group of women; however, the kind of reminder and the frequency of reminders should be carefully considered accordingly to the target group.

## Background

Gestational diabetes mellitus (GDM) is an increasing health concern in many middle-and high income countries and is mainly associated with the increasing prevalence of overweight and obesity. Worldwide, it is estimated that 16 % of live births in 2013 were complicated by hyperglycemia during pregnancy [1]. It is crucial for women with GDM to maintain a tight control of blood glucose level throughout the pregnancy to avoid complications such as macrosomia, preeclampsia or perinatal mortality [1]. However, also after giving birth the blood glucose level has to be monitored. From a meta-analysis it was found that women with prior gestational diabetes had a seven-fold risk of developing type 2 diabetes within 5 years after giving birth [2]. The progression from being at risk for type 2 diabetes (T2D) to overt diabetes can be prevented or postponed by life style changes that include a healthy diet and regular physical activity, accompanied by weight loss if the women are overweight or obese [3]. To prevent type 2 diabetes and to ensure early detection of a potential developing glucose metabolism dysfunction, recurrent screening of the women should accompany the life style changes initiated to prevent hyper-glycemia during the GDM complicated pregnancy. The American Diabetes Association and other health authorities recommend the first postpartum screening for type 2 diabetes between 6 and 12 weeks after delivery followed by screening every 3 years [4, 5]. A questionnaire-based survey in Canada has shown that both health professionals and the postpartum women were aware of the importance of postpartum screening for type 2 diabetes [6]; nevertheless, other studies report low screening rates for postpartum screening in this high risk group of women [7, 8]. In screening trials and follow-up studies of postpartum women it is well recognized that barriers and constraints for participation in screening are the main factors to eliminate if screening rates are to be increased [6, 9]. In a review of Nielsen et al., emotional stress, time pressure, difficulties with adjustment to motherhood, and loss of requisition for screening were reported as barriers among the women for postpartum screening [9]. These barriers are patient perspectives; nevertheless, health professionals’ perspectives of barriers are not yet investigated though relevant to target if screening rates should be increased: Reminder systems have shown to be a potential strategy also for improvement of health professionals’ behaviour and to improve the health care process [10]. Pierce et al. reported in their study among British primary and secondary care health professionals that 75 % had an installed reminder system to recall women to ensure postpartum screening [11]. Reminder systems; both electronic alerts for the health professional and letter or telephone reminders for the patient, are a known strategy for patient compliance improvement and have been evaluated for other outcomes than diabetes [10, 12, 13].

In this systematic review, we aimed to study the effect of implemented reminder systems for postpartum type 2 diabetes screening in the health care system where the reminders targeted either the health care professional and/or the postpartum women. The effect was measured as either the percentage of women who underwent an oral glucose tolerance test (OGTT) or other screenings tests for diabetes, or various secondary outcomes, such as response rates after the reminder, or attendance of any kind of follow-up visit.

## Methods

### Search strategy

We searched the Pubmed, Cinahl, Cochrane Review Database, and the Embase database for relevant studies. The search was performed from February to March 2014 and was limited to include only English language literature published from 2004 to 2014. Before the literature search we settled for three criteria on which literature was included. Our main inclusion criterion was in relation to the study design: We preferred intervention studies; however, we allowed studies of observational design as well to be included if an effect of a reminder system could be measured or evaluated. Our second criterion was in relation to the measured outcome: We were mainly interested in the rate of women undergoing a postpartum OGTT; however, we included other secondary outcomes; other postpartum glucose test, or rate of any kind of response from the postpartum women after the reminder was sent. Our third criterion was regarding the definition of a reminder: Reminders were defined as postal reminder, email reminders, or telephone calls/text messages for the patients. For health professionals we defined reminders as for the patients and additional the option of pop-up electronically implemented reminders/alerts or simple reminders either in paper form posted on medical reports or implemented electronically in the patient registry system. No inclusion criteria regarding the content of the reminders, since we were interested in the effect seen after reminders were sent and not necessarily received.

Clinical practice and guidelines for postpartum screening from the American Diabetes Association recommend the first early screening should be around the first 6–12 weeks postpartum, and every 1–3 years thereafter [4]. Thus, we were interested in both long term and early follow-up and compliance to screening. We defined the early term follow-up as the first postpartum follow-up visit, which is usually within the first 6 months after giving birth. Long term follow-up was defined as the following visits, usually 1 year after birth. According to this definition, we distinguish between the reminders related to the early follow-up visit, and the following reminders related to long-term follow-up no matter if the reminders sent after 12 months are the first reminders. In this way, women could miss the early follow-up but be reminded of the importance of follow-up and therefore attend a long-term follow-up visit.

**Fig. 1:**
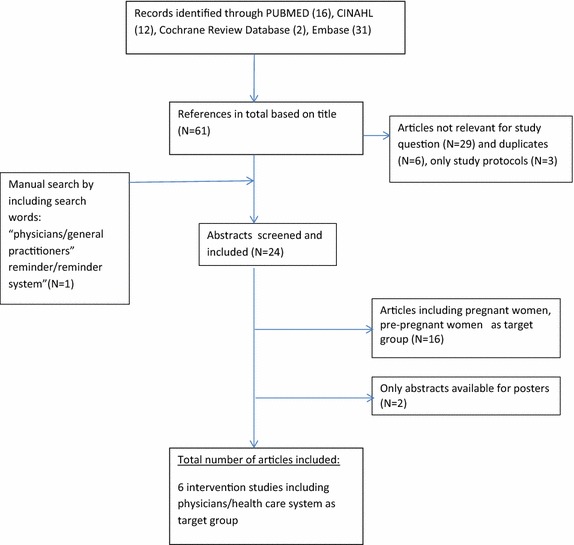
Literature search strategy

According to the PRISMA guidelines the literature extraction was divided into four stages [14]: In stage one, the search strategy was initiated by the combination of three search words: “gestational diabetes*” AND “reminder*” AND “postpartum follow-up” OR “intervention”. From that search we found 61 articles. From titles we excluded 29 articles, which were not relevant for the study question. After the database search the reference lists were further investigated in order to identify additional literature of interest. If only abstracts were available, the reference was disregarded. In the final stage, the articles were chosen after a careful reading of abstract and method sections. Figure 1 shows the flow chart for the literature search for reviewing reminder system effect. Literature search was conducted by CJ and verified by an authorized librarian trained in scientific literature search.

### Data extraction

The included studies shown in Table 1 targeted healthcare professionals, patients, or both. The studies could be either observational or experimental in design: Observational studies in the meaning of studies not directly intervening but rather studying the effect of introducing a reminder system. Intervention studies were characterised as interventions targeting a specific defined target group. We found three study protocols which we discarded, though the studies would have been relevant if results were presented. Two articles were found relevant; however, only the abstract were available and published for poster presentations. We did not make contact to the authors. We ended up with the final six references that met the inclusion criteria and were included in the review.

**Table 1:** Included intervention and observational studies for postpartum follow-up after gestational diabetes. (N = 6)

Author (year) country	Design	Setting	Reminder targeted participants and study location	Outcome and Follow-up	Description of intervention	Results
Chittleborough et al. (2010) Australia	Intervention using letter	Secondary The Diabetes Center at The Queen Elizabeth Hospital	Intervention target group: Patient and physicians 429 Postpartum women and their GPs in the GDM Recall Register receiving reminders for postpartum OGTT testing	Outcomes 1. Rate of returned update forms that was included in the reminder letter 2. The range of women reporting they had attended an OGTT test the previous year	Two times reminder letter to both patients and GPs. First time 15 months after delivery Second time every 12 months thereafter	Of the 429 women receiving a reminder letter 46 % returned an update form and 56 % had an OGTT in the previous 12 months Second reminder letter resulted in 45 % return of which 75 % had OGTT the previous 12 months
Clark et al. (2009) Canada	Intervention using letters	Secondary Ottawa Hospital, Ontario	Intervention target group: Patient and physicians 256 Patients and their physicians assigned randomly to 4 groups according to reminder intervention	Outcomes 1. Percentage of women who underwent OGTT 2. Performance of postpartum screening test	Patients assigned randomly to 4 groups: 1. Reminders both to physicians and patient 2. Reminders to patients only 3. Reminders to physician only 4 No reminders sent	OGTT rates 1. Both patient and physician reminders: 60.5 % 2. Patient only: 55.3 % Physician only: 51.6 % No reminders: 14.3 %
Korpo-Hyövälti et al. (2012) Finland	Intervention using telephone calls	Primary South Ostrobothnia, Finland	Intervention target group: Patient and physicians Counselling during pregnancy and lifestyle intervention for GDM patients. From this population a postpartum intervention was carried out among 266 high-risk-for-GDM women and their physicians in four municipalities	Outcome: the prevalence of high-risk-for-GDM women who underwent an OGTT in the postpartum period	Postpartum intervention was offered to the high-risk-for-GDM women who were included in lifestyle interventions Nurses received a list of patients and were advices to call the women for reminding them of glucose test. Reminder was sent out to the patients from the central hospital nurses or from the central hospital to the health care provider of the patient	A telephone reminder from the central hospital to the women or to their health care provider vs. no reminder: OR: 13.4, 95 % CI (2.1; 12.2), p < 0.0001
Lega et al. (2011) Canada	Register-based observational study	Secondary Toronto, Canada	Intervention target group: Patients Retrospective study of 314 women from an endocrine clinic, 173 had a checklist for the physician at their record which resulted in a reminder follow-up visit	Outcomes: OGTT Follow-up visit: The effect of a reminder system in the database on screening rates on outpatient postpartum women	No direct intervention Retrospective recording of checklist on patients in database	Intervention-group: OR (95 % CI) 2.99 (1.84; 4.85) for completion of OGTT. Intervention-group OR (95 % CI) 3.71 (2.26; 6.11) for follow-up visit
Shea et al. (2011) Canada	Intervention using mailed reminders	Secondary Ontario, Canada	Intervention group: Patients in screening sites. Three clinical sites providing screening tests with 262 patients in total: two sites received reminder (A and B), one did not (C)	Outcome: Percentage of women who received an OGTT test within 6 months after delivery	Intervention targeted patients Clinical site A received mailed reminders with a laboratory requisition for OGTT Clinical site B were sent a laboratory requisition for OGTT, phoned for appointment, or both Clinical site C: no reminders	Prediction of postpartum glucose screening: reminder site A vs site C: OR (95 % CI): 1.57 (0.66; 3.70) reminder site B vs. site C: OR (95 % CI): 3.10 (1.35; 7.14)
Vesco et al. (2012) US	Intervention using combined telephone calls/emails and staff education	Secondary Oregon and Washington	Intervention target group: Patients 200 Women for the pre-implementation (no intervention group) 179 Women for the post-implementation (intervention group)	Two-fold outcome 1. Outcome among providers was the rates of women for whom a screening test was ordered 2. Patient outcome measured by postpartum screening rates among previous GDM patients	Implementation of a process improvement program consisting of 1. Staff education sessions 2. Revised GDM protocols 3. Implementation of system-based reminders to call in women for glucose screening send out within 3 months of delivery and after 3 months of delivery	With implementation of program the rate of women receiving order for OGTT increased from 77.5 to 88.8 % Final screening rate with completion of OGTT increased from 59.5 to 71.5 % (p = 0.004), (HR (95 % CI) 1.37 (1.07; 1.75)

## Results

### Search results

The included studies were published from 2009 to 2012 and were conducted in Finland (1), Australia (1), United States (1), and Canada (3). Two studies focused on the long term postpartum screening: One study investigated the effect of reminders sent 15 months after delivery and every 12 months thereafter [15] where women according to recommendation should have attended the first screening consultation and Vesco et al. investigated the effect of reminders sent before and after 3 months after delivery [16]. However, all studies were conducted aiming at reminding of the first early postpartum glucose intolerance screening test. Ethical approval for this literature study was not required.

### Population characteristics

Study populations differed between the studies. The effect of reminder systems were studied on patients only (N = 3), and on both patients and health professionals (N = 3). Health professionals included general practitioners, endocrinologists, and medical residents [17, 18]. Only one study reported age of the health professionals, and found 83.8 % were above 30 years of age [18]. In the three studies including patient reminders only, the patients were randomized according to a specific setting (clinical site being the intervention group) [19], patients belonging to a specific municipality [20], or patients were already included in a registry allocated to a clinical site (hospitals) or joint treatment organization [15, 16]. The studies reported the women’s age as mean or percentage under and over 30 or 35 years as cut-off points. Mean age (SD) ranged from 30.1 (±5.7) [20] to 34.9 (±5.2) [17]. In the papers reporting age ranges 72.8 % were over 30 years of age in Clark et al. [18] and in Vesco et al. [16] 30 and 36.3 % in the pre-implementation of reminder system group and post-implementation of the reminder system group, respectively, were above 35 years of age. Only one study did not report the women’s age [15].

### Reminder-systems

In the six studies included, the reminder-systems were either the main intervention [15, 18–20] or an integrated part of a larger intervention program [16]. In the study of Vesco et al. the postal reminders for patients was a part of a larger process improvement program that also included revised nursing protocols for the GDM women, enhancing the electronic medical record system, and education of the clinical staff in addition to the postal patient reminders [16]. The results of Vesco et al. were not divided into differentiated effects of the various initiatives and therefore the results are included in the patient reminder group. The health professionals’ reminders were based on system-based pop-up messages reminding the professional to recall women for postpartum check-up. The patient reminders varied from telephone calls from nurses to the patient [16, 20] or letters or mailed reminders sent to the patient [18, 19].

### Physician interventions

The primary sector and secondary sector are joint in the health care system by interaction around the patient; however, with different responsibilities and duties. In our definition, primary sector also include health care staff at elderly centres, pharmacies, and infant and neonatal health care providers for supporting the mother. General practitioners, as being the primary player in the primary sector, have first contact with citizens. Thus, the primary sector can in addition to being the first contact with patients also play a health promoting and disease preventive role towards the public. The secondary sector includes hospitals and specialised health care providers. The secondary sectors is responsible for the complex treatment of patients and the secondary sector can normally only be contacted by patients through a first contact to the primary sector. Going through the literature we therefor made a distinction among the health care professionals. Primary health providers are general practitioners since they act as a first point of consultation for the women with former gestational diabetes, however, secondary system play the dominating role treating the women during the pregnancy and preventing a worsening of the GDM state.

The occurrence of using reminder systems for recalling postpartum women for glucose intolerance screening have been studied before [21]. Nonetheless, diverse results on the use of reminder systems exist: One study found that 8.2 % used an electronic alert or reminder to call women in for postpartum screening, but only 12.8 % of the 306 health professionals actually sent reminders to patients when it was time for postpartum screening [21]. Another study reported that 75 % of the secondary care specialists (obstetricians and gynaecologists) had an integrated system in place to alert when postpartum women failed to attend their follow-up visit. Among these secondary care providers 73 % sent a notification to the general practitioners (GP), proposing the GP to recall the women for follow-up [11].

Among the primary care GPs 39 % recalled women on an annual basis and 35 % advised the women to attend screening in the future. The perception of responsibility seems to be a factor affecting the recall of patients: 45 % of the GPs thought it was the responsibility of the primary care sector to conduct early follow-up on previous GDM women; however, 26 % of the GPs thought there was no clear responsibility in any sector [11]. With the above results in mind, it is interesting to evaluate the effect reminders have on physicians and health professionals and the effect reminders have on screening rates of postpartum women.

We included two intervention studies with the focus on health professional alerts for recalling postpartum women: Clark et al. made a 2*2 factorial randomized control design on patient and/or physician reminder interventions. The study included 256 eligible women and their 256 physicians. The randomized groups were designed as: physician reminders only; patient reminders only; both physician and patient reminders; and no reminders (controls). The primary outcome of the study, as seen in Table 1, was the proportion of women who underwent an OGTT within 1 year after delivery and the reminder was sent approximately 3 months after delivery in order to conform with screening recommendations. Secondary outcome was measured as the performance of any kind of screening test. When only physicians were reminded, the postpartum women were more likely to undergo an OGTT [OR 8.4 (95 % CI 2.4; 28.5)] than if no reminders were sent (the control group). Interestingly, the association with compliance to screening decreased if both the physician and the patient were reminded OR 5.2 (95 %CI 1.4; 19.6) compared to controls. However, the results should be interpreted carefully since the study populations of both patients and physicians were small: after loss to follow-up 112 physicians remained for analyses. The number of women in the three groups varied from 31 (physician reminders only) to 81 (both physician and patient reminders). Loss to follow-up was mainly due to movement of either patient or physician. This was taken into account in sensitivity analyses where the study assumed that patients lost to follow-up all underwent OGTT screening (analyses 1) or did not undergo OGTT screening (analyses 2). When assuming that none of the women lost to follow-up underwent OGTT the effect of the reminders to both patients and physicians and the interventions on physicians only remained significant, OR 3.9 (95 % CI 1.1; 13.9) and OR 4.8 (95 % CI 1.6; 14.9), respectively.

In Lega et al. a retrospective observational study of a checklist procedure was evaluated. The study included 314 postpartum women with previous GDM from an internal hospital record. From this study population 173 of the women were enrolled with a checklist depicting to the health professionals whether the woman had attended her postpartum visit. The study was an evaluation of the effect of implementing this checklist procedure in the register. Therefore no direct intervention took place; however, having a checklist registered was shown to be associated with completed OGTT between 6 weeks and 6 months postpartum by OR 2.99 (95 % CI 1.84; 4.85). As for the study of Clark et al., this study also had secondary outcomes measured as the rate of women attending a postpartum visit, not necessary completing an OGTT. The registration of a checklist for the patient was positively associated with attending a postpartum visit within the same time frame as for OGTT: OR 3.71 (95 % CI 2.26; 6.11). Since no direct intervention took place, a major strength of the study was no loss to follow-up and the directly effect on the registration of the patients the effect of a checklist targeting the attention of the health professional. Nevertheless, the study did not discuss differences in the allocation of a checklist and if the allocation could confound the results in any way. Both the study of Lega et al. and Clark et al. had the outcome of increasing the first screening after birth (early term screening) meaning undergoing an OGTT within 6 months after delivery.

Based on these two studies it seems plausible that reminders for health professionals can increase the postpartum OGTT screening among women with previous GDM. However, this conclusion is based on two studies only, where one study had a large loss to follow-up and the large differences in the number of intervention groups. Furthermore, the conclusion can only be drawn on the first early screening after delivery and not on a long term basis. Furthermore, interestingly it seems that reminding both patient and physician does not result in increased screening rates compared to reminding either patients or physicians.

### Patient interventions

The most recent study on patients’ perception of health professionals’ responsibility for postpartum screening revealed that primary care providers, such as general practitioners, were responsible for the recall of women for postpartum OGTT. Out of 136 postpartum patients 76 % thought it was the GP, whereas 8.8 % thought it was the responsibility of the obstetrician only, and 11.8 % thought it was the health professional treating them for gestational diabetes [6]. This confusion of which health professional to turn to for postpartum diabetes screening could potentially influence screening rates among postpartum patients in some countries. Furthermore, a qualitative study conducted in the Johns Hopkins Bayview Medical Center’s obstetric clinical practice revealed that receiving care from multiple providers and lack of continuity of care were barriers for women’s participation in postpartum screening [22].

Reminder-systems for patients could provide this continuity of care but also a continuity of the awareness of the risk for development of diabetes. In the study of Shea et al. two clinical sites were allocated to have implemented reminder systems contacting the postpartum patients by telephone or letter [19]. Despite that the patient groups had a large variation in number of patients (control site: 117, site A: 90, site B: 55) 28 % of patients, when adding site A and B together, returned for a postpartum visit and completed an OGTT test compared to 13.75 % (p = 0.01) from the control site. In this intervention site A and B differed: In site A only laboratory requisitions and reminder letters were sent. In site B reminder letters were sent, and an additional telephone call was made. This difference in reminder approach resulted in a stronger association of site B with completion of OGTT: OR 3.10 (95 % CI 1.35; 7.14) compared to the non-reminder control site whereas site A had an OR 1.57 (95 % CI 0.66; 3.70) compared to the control site. When the outcome was set as completion of any kind of screening test including HbA1c, random glucose test, or fasting glucose test the difference in association between site A and B attenuated: OR: 1.09 (95 % CI 0.56; 2.13) and OR 1.33 (95 % CI 0.65; 2.71) for site A vs control site and site B vs. control site, respectively [19]. In Korpi-Hyövälti et al. a similar reminder approach was implemented: High risk-of-GDM women were called by health care nurses encouraging the women to complete a visit for an OGTT test. Of the women who completed an OGTT 83.2 % had received a phone call compared to 49.1 % completing OGTT in the group of women not receiving phone reminders [20].

The potential of personal approach reminders, such as phone call, is significant and a personal approach can have a large effect whereas letters and emails that can be perceived less personal and committing to the patients still have an effect on screening rates; nonetheless, they are less effective than phone calls. This is in consistence with qualitative research findings; a personal approach from medical staff to the patient enhances the commitment of the patient [22].

The number of reminders is another factor influencing screening rates. In Chittleborough et al. 817 postpartum women registered in the Gestational Diabetes Mellitus Recall Register allocated at three local hospitals received up to 6 reminders over a period of 6 years. The main outcome was the return of an update form and second, the information of completion of an OGTT the previous 12 months. The proportion of women who returned the update form declined over time; however, the percentage of women who reported to have an OGTT completed remained high: 56.3 % of 429 women after the first reminder letter and 66.7 % of 26 women after the sixth reminder letter. In Vesco et al. it was reported that an implementation of staff education combined with patient reminders that included telephone call, email or letter, and in-person reminder in 7 clinics handling postpartum follow-up visits increased screening rates significantly. After the implementation, cox regression analysis revealed a HR 1.37 (95 % CI 1.10; 1.70) for completion of screening. Screening was defined as completed OGTT, or measured fasting glucose. The number of reminders influenced the completion rate: after the first reminder 80 % of the women completed a glucose test (either fasting or OGTT). According to the authors 41 % of the remaining completed after second reminder and after the third reminder 28 % of the women completed the ordered glucose test [16].

### Staff education and awareness in combination with reminder systems

In the study of Vesco et al. the reminder system was combined with implementation of an educational intervention among the health staff [16]. The study found that the implementation of staff education has obviously influenced awareness of reminding women to undergo screening. The largest effect on the amount of reminders send was seen on a long-term aspect where the proportion of any kind of the three mentioned reminders increased from 27 to 59 % (p < 0.0001). This is not surprising since other studies have found that integrated study protocols that help the health professional will improve keeping focus on and prioritize high risk patients: Ko et al. reported that more than half of their study population of obstetricians and gynaecologist (N = 306) reported to have clinical guidelines addressing postpartum screening integrated in their practice. Clinical guidelines were more widespread in clinics of frequent screeners. If the obstetricians and gynaecologists reported to screen the women “always” or “most of the time” the authors’ defined the health professional to be “frequent screener”. Likewise, health professionals who reported “sometimes”, “rarely” or “never” to conduct screening were characterised as “infrequent screeners” [21]. Furthermore, the obstetricians and gynaecologists identified as frequent postpartum screeners were more likely to consider screening a priority in their practice (25.5 % for frequent screeners vs. 13.3 % for non-frequent screeners, p < 0.001) [21]. In line with this, Hunsberger et al. conducted a mail survey among family medicine physicians and specialized physicians in obstetric or gynaecology. They found that postpartum screening rates depended on whether it was a priority of the physician or clinic (OR 4.39, 95 % CI 1.69–7.94) and mostly if the physician thought screening was the norm among this special group of patients [OR 3.66, 95 % CI (1.65–11.69)]. However, the study had a low response rate of only 42 % in total (N = 285) and 58 % (N = 166) of these were family physicians compared to 42 % (N = 119) obstetricians or gynaecologists [23].

## Discussion

In this systematic review we studied the effect of reminders for health care professionals and/or postpartum women with previous GDM or reminder systems integrated in secondary or primary care. For the patient reminders it seems that personal reminders have a better effect on the screening rates and Chittleborough et al. showed that even though the response rate from each reminder sent declined—the proportion of women completing OGTT over time remained high. Furthermore, patient reminder systems can with benefit be combined with educational interventions among the health professionals to increase awareness of the importance of using the reminder system.

A recent review on barriers for gestational diabetes screening and barriers for the subsequent postpartum screening revealed that time and the new role as a mother were the two main barriers among patients [9]. However, few studies have so far been interested in the barriers that may exist among health professional conducting screening test among this high risk for diabetes patient group. Two studies only had the main aim of their questionnaire surveys to study barriers among health professionals: Stuebe et al. identified various barriers among the participating physicians: poor communication between primary care physicians and secondary care specialists, and poor documentation in the electronic record of gestational diabetes (filed for 45.8 % of women only), even though this was recommended [24]. In Keeley et al., the barriers for not screening patients was that the physician did not have consultations with the patient. This was reported by 37 % of the 63 physicians that did not screen their patients. The second most common barrier for not screening was reported by 33 % and was due to non-arrangement of the test, even though there has been a consultation regarding other health problems. Lack of awareness regarding the screening issue between health professionals and between health professional and patient could be solved by integrated reminder systems alerting the professionals either to contact other professionals or to call in women for screening. Integrated reminder systems could automatically send out reminders for patients as well.

The use of reminder systems is not a new intervention. Randomized controlled trials have evaluated the effect of reminder alerts for physicians in order to increase screening rates for colorectal cancer [25], mammography [26], and breast- and cervical cancer [27]. These studies provide evidence for the improvement that implemented reminder systems can give for these specific diseases. However, it does not mean it will be effective for increasing postpartum screening rates but definitely leave potentials for trials to replicate interventions in order to find suitable reminder systems for health professionals handling postpartum testing of women. One study found significantly higher screenings rates for deficiencies, hypertension and diabetes among elderly patients in an ordinary primary care setting in Stockholm, Sweden where a reminder system had been implemented among GPs. The study found significantly higher rates of systolic blood pressure, and cobalamin deficiency, whereas the rates of diabetes and anaemia were non-significantly increased among elderly patients of the GPS in the intervention group. The authors concluded that implementation of this reminder system in a primary care setting [28] could have similar benefits in a secondary sector setting. It was in an updated Cochrane Review concluded that mobile phone text messaging reminders increased attendance at healthcare appointments and this was compared to no reminders, or postal reminders [12].

One could speculate on the effectiveness of the reminders. The effect of reminders and implementations of reminder systems for either health professionals or patients could be improved by increasing the amount of information e.g. by patient specific campaigns, on the importance of postpartum screening and the awareness of the risk of developing type 2 diabetes.

### Strengths and limitations

We have several limitations in our study. The most obvious is the lack of controlled randomised intervention studies. A similar systematic review investigating reminders for women with previous GDM included randomised controlled trials only [29]. This systematic review ended up with only one study in their analyses. In our opinion, other studies not necessary of interventional design can provide valuable information as well. We therefore broadened our search to include non-randomised studies and observational studies. However, with the lack of randomised controlled trials we are not able to study negative results of reminder systems or studies showing no effect. Another large limitation is the lack of long-term follow-up studies: four out of six studies focused on early term follow-up within the first year after giving birth. This leaves us with scarce results and unable to make solid conclusions for a long-term effect of reminders for postpartum screening. Finally, we were able to find one study focusing on primary care health setting, whereas the other five focused on secondary health care setting. A large diversity between countries variations exist when it comes to postpartum follow-up visits. In Denmark, women are encouraged to attend their postpartum diabetes screening at their general practitioner.

Nevertheless, a strength of our study is the variation in type of reminders used. Effect of letters, telephone calls or emails for patients, and reminders attached to medical files or register-based reminders to health professionals were included in the studies. Despite the type of reminder—all reminders had an effect. This is important seen from a general point of view: The use of reminders and reminder systems has one aim: to increase awareness of the importance of attending the recommended postpartum diabetes screening. From a patient point of view awareness on the risk of developing diabetes has to be maintained. From the health professional’s point of view reminder systems help to keep track on patients and rates of follow-up. Therefore, we see it as an important finding, that all the articles showed an effect despite type of reminder.

## Conclusions

The included studies have shown that both reminder systems to patients and to health professionals are successful in increasing postpartum screening rates. Nevertheless, screening rates and effectiveness have to be differentiated in early and long term effects postpartum. Evidence is scarce for long term effects past the first follow-up visit or with visits placed after the first year postpartum. The number of reminders sent to patients and the kind of reminder also influence the effectiveness in a way that personal confrontation reminders such as telephone calls are more effective than letters. For the number of reminders evidence is lacking, however, it seems that with numerous reminders more women will undergo a follow-up visit over time; nevertheless, some women will not attend or will refuse to attend.

Due to lack of evidence, future research could focus on longer term compliance to screening among women with previous GDM to see the effect of reminder systems ideally both for health professionals and for patients. One could speculate, whether postpartum health communication could be linked to health examinations of the child when the woman is at the general practitioner. In this way, pop-up reminders linking child examination and postpartum examination would benefit the awareness among the professionals and patients, and by that mean, solve one of the main barriers identified among health professionals [9]: lack of awareness and contact to the patient, and inadequate knowledge exchange among professionals.

## Abbreviations

GDM: gestational diabetes mellitus
T2D: type 2 diabetes
OGTT: oral glucose tolerance test
GP: general practitioner
